# Supplementary material for: Clinical Evidence of Mesenchymal Stromal Cells for Cerebral Palsy: Scoping Review with Meta-Analysis of Efficacy in Gross Motor Outcomes
Source: Cells. 2025 May 12;14(10):700. doi: 10.3390/cells14100700 (PMC12110704; doi:10.3390/cells14100700)
Supplement: Supplementary file 1 [file cells-14-00700-s001.zip › Table S2_Search Strategy_28th March 2025.pdf]

Table S2: Search Strategy

**Search terms**
1. MEDLINE, Cochrane Central and Embase via Ovid
“cerebral palsy”.mp [mp=ti, ab, hw, kw, tn, ot, dm, mf, dv, kf, fx, dq, bt, nm, ox, px, rx, an, ui, sy, ux, mx, sh] ***AND***
“mesenchymal”.mp [mp=ti, ab, hw, kw, tn, ot, dm, mf, dv, kf, fx, dq, bt, nm, ox, px, rx, an, ui, sy, ux, mx, sh]
2. Google Scholar
“cerebral palsy mesenchymal”
3. ClinicalTrials.gov
Searched on “condition”: “cerebral palsy”
Searched on “intervention”: “mesenchymal”
4. Japan University hospital Medical Information Network Clinical Trial Registry (UMIN-CTR)
5. Japan Medical Association Clinical Trial Registry (JMA-CTR)
6. EU Clinical Trials Register (EudraCT)
7. Chinese Clinical Trial Registry (ChiCTR)
8. WHO International Clinical Trials Registry Platform (ICTRP)
9. Netherlands Trial Register (NTR)
10. Australian New Zealand Clinical Trial Registry (ANZCTR)
11. Clinical Trials Registry-India (CTRI)
12. German Clinical Trials Register (DRKS)
13. Iranian Registry of Clinical Trials (IRCT)
14. Clinical Research Information Service from South Korea (CRiS)
Searched on general search function or by “condition” or “disease” or “public title” fields: “cerebral palsy”
