# Supplementary material for: Clinical Evidence of Mesenchymal Stromal Cells for Cerebral Palsy: Scoping Review with Meta-Analysis of Efficacy in Gross Motor Outcomes
Source: Cells. 2025 May 12;14(10):700. doi: 10.3390/cells14100700 (PMC12110704; doi:10.3390/cells14100700)
Supplement: Supplementary file 1 [file cells-14-00700-s001.zip › Table S3_Summary of Included Reports_28th March 2025.pdf]

Table S3: Summary of Included Reports

	Study information			Participant information			MSC information	
	**Author, Year, Reference/ Trial Registration**	**[a]Report type**	**[b]Status**	**Participants treated/total**	**CP type/ topography/ severity**	**Age**	**Source**	**Route, dose, # treatments**
	**(a) PUBLISHED REPORTS**							
1.	Chen 2011 [29]	Phase 2	Published	17/34	NI	6mo-4y	Auto, BM	IT, **total dose 3-5×10^6**, 1 treatment
2.	Abo Elkheir 2014 [26]	Phase 2	Published (abstract)	26/52	NI	NI	Auto, BM	IT, **NI**
3.	Gabr 2015 [34]	Phase 2	Published	44/100	Spastic, dyskinetic, NI GMFCS 2-5	1-7y	Auto, BM	IT, **2x10^6/kg body weight**[c], 2-6 treatments
4.	Liu 2017 [40] **ChiCTR-TRC-12002568**	Phase 2	Published	35/105	Spastic, NI, GMFCS 2-5	6mo-11y	Allo, BM	IT, **1x10^6/kg body weight**, 4 treatments
5.	Huang 2018 [38]	Phase 2	Published	27/56	NI	3-12y	Allo, CT	IV, **total dose 50x10^6**, 8 treatments
6.	Gu 2020 [35] **ChiCTR1800016554**	Phase 2	Published	19/39	NI	2-12y	Allo, CT	IV, **total dose 45-55 ×10^6**, 1 treatment
7.	Amanat 2021 [27] **IRCT201706176907N13/NCT03795974**	Phase 2	Published	36/72	Spastic, NI, GMFCS 2-5	4-14y	Allo, CT	IT, **total dose 20x10^6**, 1 treatment
8.	Sun 2022 [21] **NCT03473301**	Phase 2	Published	23/91	Hypertonic, all topography, GMFCS 1-4	2-5y	Allo, CT	IV, **2x10^6/kg body weight**, 3 treatments
9.	[#]Chrościńska-Kawczyk 2024 [30]	Compassionate/ Expanded Access	Published	401/401	NI, GMFCS 1-5	9mo-45y	Allo, CT	IV (and some additional IT), **total dose 10-70x10^6**, 1-13 treatments
10.	[#]Rojek 2020 [44]		Published (abstract)					
11.	Venkataramana 2011 [45]	Phase 1	Published (abstract)	18/18	NI	1-17y	NI	IC, **NI**

12.	Liu 2012 [41] **ChiCTR-TRC-11001419**	Phase 1	Published	17/29	NI	1-6y	Auto, BM	IT or IC and IT, **NI**, 4 IT treatments or 1 IC and 2 IT
13.	Wang 2013 [49]	Phase 1	Published	46/46	Spastic, dyskinetic and mixed, NI, GMFCS 1-5	6mo-15y	Auto, BM	IT or IT and intraparenchymal, **total dose 20x10^6**, 3-4 treatments
14.	Wang 2015 [50] **ChiCTR-ONC-12003124**	Phase 1	Published	16/16	Spastic, NI	3-12y	Allo, CT	IT, **total dose 10-15 x10^6**, 4 treatments
15.	Wu 2008 [52]	Case Series	Published	20/20	NI	1-10y	Allo, UCB	IT, **NI**[e], 4 treatments
16.	Wang 2010 [51]	Case Series	Published	24/24	Spastic, ataxic, dyskinetic hypotonic GMFCS 4-5	6mo-12y	Auto, BM	Intraventricular and IT, **total dose 20x10^6 or 40x10^6 respectively,** 3-4 treatments total
17.	Du 2011 [32]	Case Series	Published	50/50	Mixed, hypotonic and ataxic, GMFCS 4-5[d]	1-6y	Auto, BM	Intraventricular, **total dose 6-7x10^6,** 1 treatment
18.	Wang 2011 [47]	Case Series	Published	51/51[f]	NI	20-38y	Allo, CT	IT and IV, **NI,** 4 treatments in total
19.	Li 2012 [39]	Case Series	Published	1/1	NI	11y	Auto, BM	IV, **total dose 40x10^6**, 4 treatments
20.	Yang 2012 [53]	Case Series	Published	25/25	Spastic, hypotonic, dyskinetic, ataxic, mixed, NI	1-12y	Allo, CT	IT and IV, **total dose 20-60x10^6**, 4-6 treatments
21.	Wang 2013 [48] **ChiCTR-TNRC-10000928**	Case Series	Published	1/1	NI	5y	Allo, CT	IT and IV, **total dose 5-10x10^6**, 7 treatments
22.	Miao 2015 [42]	Case Series	Published	11/100[g]	NI	NI	Allo, CT	IT, **NI**, 4-6 treatments
23.	Zhang 2015 [5]	Case Series	Published	1/1	NI	6mo	Allo, UCB	IV, **total dose 50x10^6**, 12 treatments
24.	Dong 2018 [31]	Case Series	Published	1/1	NI	4y	Allo, CT	IT and IV, **total dose 1.625-7.0x10^6**, 3 treatments
25.	Hirano 2018 [37]	Case Series	Published	1/1	NI, hemiplegic, NI	7y	Allo, adipose	IV and IM and SC and Joint, **total dose 30-750 x10^6**, 8 treatments

26.	Okur 2018 [43]	Case Series	Published	1/1	Spastic, GMFCS 5	6y	Allo, CT	IT and IV, **1x10^6/kg body weight**, 4 treatments
27.	Fu 2019 [33]	Case series	Published	57/57	Spastic, NI, GMFCS 4-5	1mo-12y	Allo CT	IT, **total dose 10x10^6**, 4-8 treatments
28.	Vij 2023 [46]	Case Series	Published	1/1	Spastic, quadriplegic, NI	14y	Auto, adipose	IV, **total dose 28-57.1x10^6**, 32 treatments
29.	Hastuti 2023 [36]	Case Series	Published (abstract)	1/1	NI	13mo	Allo, CT	IV, **total dose 10x10^6**, 3 treatments
30.	Boyalı 2024 [28]	Case Series	Published	4/4	NI, GMFCS 4-5	1-9y	Allo, CT	IT, **IV and IM, 1 ×10^6/kg body weight**, 6 treatments
	**(b) UNPUBLISHED, REGISTERED CLINICAL TRIALS**							
31.	**NCT01929434**	Phase 3	Completed	100/300	NI	1-14y	NI, CT	IT, **NI**
32.	**IRCT20110628006907N18**	Phase 3	Completed	75/150	Spastic, diplegia, quadriplegia or hemiplegia, NI	2-14y	Allo, CT	IT, **total dose 20x10^6**, 3 treatments
33.	**IRCT20200502047277N5**	Phase 2	Completed	23/46	NI	1-18y	Auto, BM	IT, **NI** [h]
34.	**IRCT20110628006907N14**	Phase 2	Completed	35/70	Spastic, diplegia, quadriplegia or hemiplegia, GMFCS 2-4	2-14y	Allo, CT	IT, **total dose 20x10^6**, 3 treatments
35.	**NCT03979898**	Phase 1	Completed	1/1	Spastic, paraplegia or bilateral quadriplegia, NI	2-12y	Auto, adipose	IV, **5-150x10^6/kg body weight**, 1 treatment
36.	**NCT04314687**	Phase 2	Unknown status	26[i]/78	NI, quadriplegia, NI	6mo- 3y	Allo, CT	IT, **NI**
37.	**NCT03414697**	Phase 2	Unknown status	33/44	Spastic, quadriplegic, GMFCS 2-3	2-18y	Allo, CT	IT or IV or IN, **total dose >10x10^6**, 4 treatments
38.	**NCT05018819**	Phase 1	Recruiting	15/15	NI	All ages	Allo, CT	IV, **total dose 100x10^6**, 1 treatment

39.	**NCT04873752**	Phase 1	Active, not recruiting	6/6	NI, GMFCS 2-3	1-2y	Allo, CT	IV, **2.5x10^{6}/kg body weight**, 8 treatments
40.	**jRCTb040230042**	Phase 1	Active, not recruiting	3/3	NI, GMFCS 2-3	6-11y	Auto, dental pulp	IV, **NI**

[a]case series include case reports, case series and cohort studies; Phase 1 includes single-arm studies even if they investigate efficacy outcomes; Phase 2 include randomised and non-randomised controlled studies.

[b]status of unpublished clinical trials are taken directly from the corresponding trial registration information. Those listed as "Completed" have either finished recruitment or completed the study altogether.

[C]unclear how many cells given per dose. 2x10^6/kg body weight is the target total dose of all treatments

[d]Listed as *severe or extremely severe* for severity of CP

[e]3-4x10^10 cells grown in culture but not specified as dose administered

[f]report details 51 participants out of a larger cohort of 229

[g]mixed cohort study, 11 treated with CP

[h]total volume of cells reported as 1x10^{6}/ml but unknown dose

[i]Not accounting for other cell cohort receiving mixed cell therapy

[#]personal correspondence with authors from FAMICORD Group. Authors provided compiled patient and cell characteristic information and incorporated recent figures from their compassionate access program

Abbreviations: Auto, autologous; allo, allogeneic; BM, bone marrow; cord tissue, CT, IC, intracranial; IN, intranasal; IT, intrathecal; IV, intravenous; GMFCS, Gross Motor Functional Classification System; mo, month; NI, no information; UCB, umbilical cord blood; y, year.
