# Supplementary material for: Clinical Evidence of Mesenchymal Stromal Cells for Cerebral Palsy: Scoping Review with Meta-Analysis of Efficacy in Gross Motor Outcomes
Source: Cells. 2025 May 12;14(10):700. doi: 10.3390/cells14100700 (PMC12110704; doi:10.3390/cells14100700)
Supplement: Supplementary file 1 [file cells-14-00700-s001.zip › Table S4_Outcome Measures for MA_28th March 2025.pdf]

Table S4: Motor Function Outcomes and Timepoints

Study	Details of gross motor function measure	Timepoints and presentation of results
Huang 2018 [38]	GMFM-88<br>CFA	Baseline, 3, 6, 12, 24 months<br>**Mean change from baseline**
Gu 2020 [35]	GMFM-88<br>CFA	Baseline, 1, 3, 6, 12 months<br>**Mean change from baseline**
Liu 2012 [41]	GMFM (version not specified)	Baseline, 3, 6, 12 months<br>**Total scores only**
Amanat 2021 [27]	GMFM-66<br>Modified Ashworth Scale<br>PEDI	Baseline, 1, 3, 6, 12 months<br>**Mean change from baseline and total scores**
Sun 2022 [21]	GMFM-66	Baseline, 6, 12 months<br>**Estimated change from baseline**
Chen 2011 [29]	GMFM-66	Baseline, 3 months<br>**Mean change from baseline**
Abo Elkheir 2014 [26]	Boyd's Developmental Progress Scales	Baseline, 12 months
Gabr 2015 [34]	GMFCS, PEDI	Baseline, 12 months

Abbreviations: CFA, Comprehensive Functional Assessment; GMFM, Gross Motor Function Measure; PEDI, Pediatric Evaluation of Disability Inventory.
